# Supplementary material for: Impaired envelope integrity in the absence of SanA is linked to increased lipid II availability and an imbalance of septal peptidoglycan synthesis
Source: mBio. 2026 Apr 30;17(6):e00635-26. doi: 10.1128/mbio.00635-26 (PMC13251401; doi:10.1128/mbio.00635-26)
Supplement: Supplemental Material — Supplemental figures and tables. [file mbio.00635-26-s0001.pdf]

Supplemental Material For:

Impaired envelope integrity in the absence of SanA is linked to increased lipid II availability and an imbalance of septal peptidoglycan synthesis

Joseph F. Carr[a], Carolina Basurto De Santiago[a,‡], Shivani A. Bhut[a,‡], Daniel J. Warzecha[a,‡], Sarah A. Vastani[a], Robert Wei[a], Carmen M. Herrera[b], M. Stephen Trent[b,c], Beiyan Nan[a], Angela M. Mitchell[a,#]

[a] Department of Biology, Texas A&M University, College Station, Texas, USA

[b] Department of Infectious Diseases, College of Veterinary Medicine, University of Georgia, Athens, Georgia, USA

[c] Department of Microbiology, College of Art and Sciences; University of Georgia, Athens, Georgia, USA

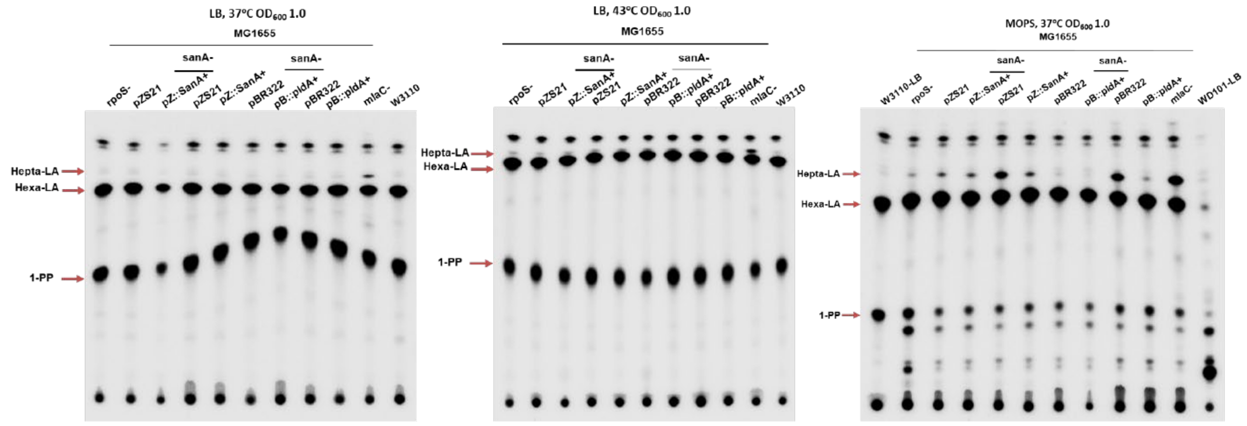

Figure S1: Loss of SanA causes accumulation of hepta-acylated lipid A when cells are grown with glucose as a carbon source. Full TLC images are shown for the experiment shown in Figure 1D.

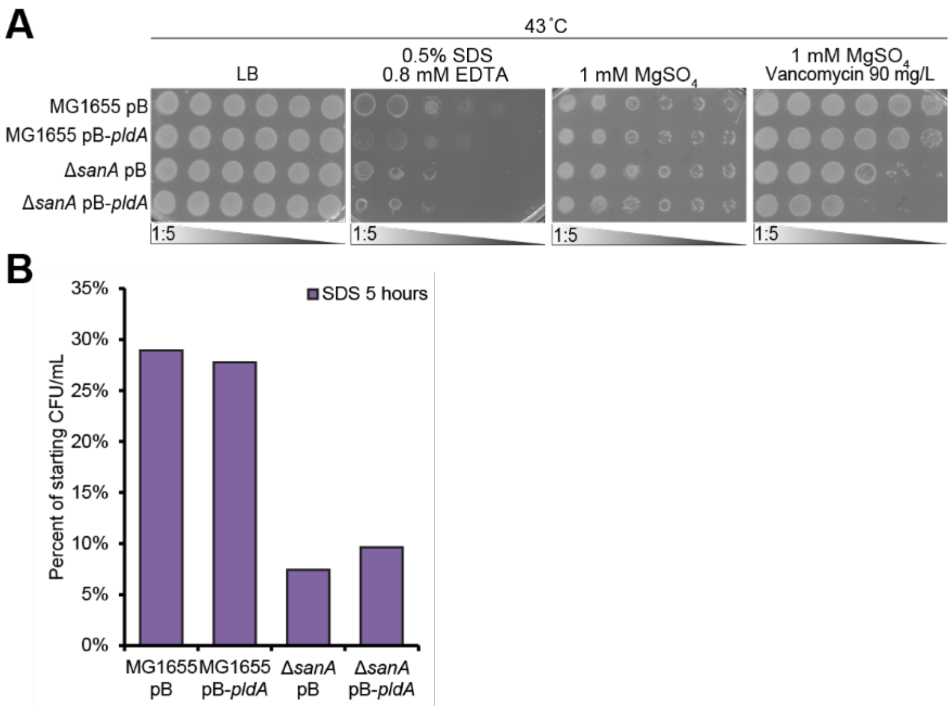

Figure S2: Overexpression of *pldA* does not suppress the envelope permeability phenotypes caused by loss of SanA. (A) The effect of *pldA* overexpression on the SDS-EDTA resistance and vancomycin resistance of a Δ*sanA* strain at 43 °C was assayed by EOP. Overexpression of *pldA* did not suppress these phenotypes. **(B)** The viability of the indicated strains was assayed after 5 hours of SDS treatment during carbon-limitation stationary phase. Overexpression of *pldA* did not restore the SDS resistance of the Δ*sanA* strain.

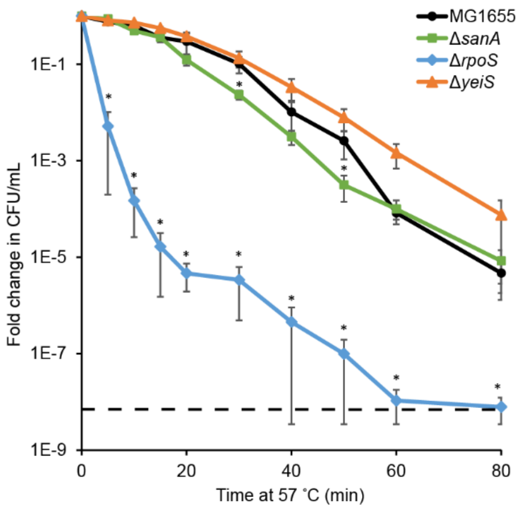

Figure S3: Temperature sensitivity caused by loss of RpoS is greater than that caused by loss of SanA. The Δ*sanA* strain grown to carbon limitation has decreased heat shock survival at 57 °C compared to wild type, but less so than an RpoS mutant. This is not true of *sanA* operon partner *yeiS* which does not show temperature sensitivity. Data for the wild type and Δ*sanA* strains are the same as is shown in Figure 1E. Data are the average of 3 to 4 biological replicates ± the SEM. * $p<0.05$ by the Mann Whitney Test.

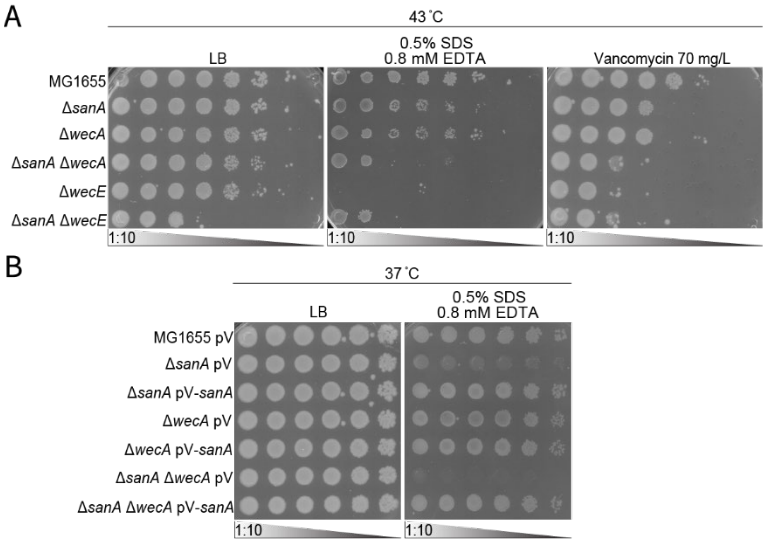

Figure S4: *sanA* has genetic interactions with mutations in ECA synthesis. (A) The SDS-EDTA sensitivity of a Δ*sanA* Δ*wecA* strain is maintained at 43 °C; however, a vancomycin phenotype is less apparent. **(B)** The SDS-EDTA sensitivity of a Δ*sanA* Δ*wecA* strain can be complemented by plasmid-based expression of *sanA*. **(C)** OD_{600} growth curve corresponding to the reporter assay in Figure 2C. Data are shown as the average of three biological replicates ± the SEM.

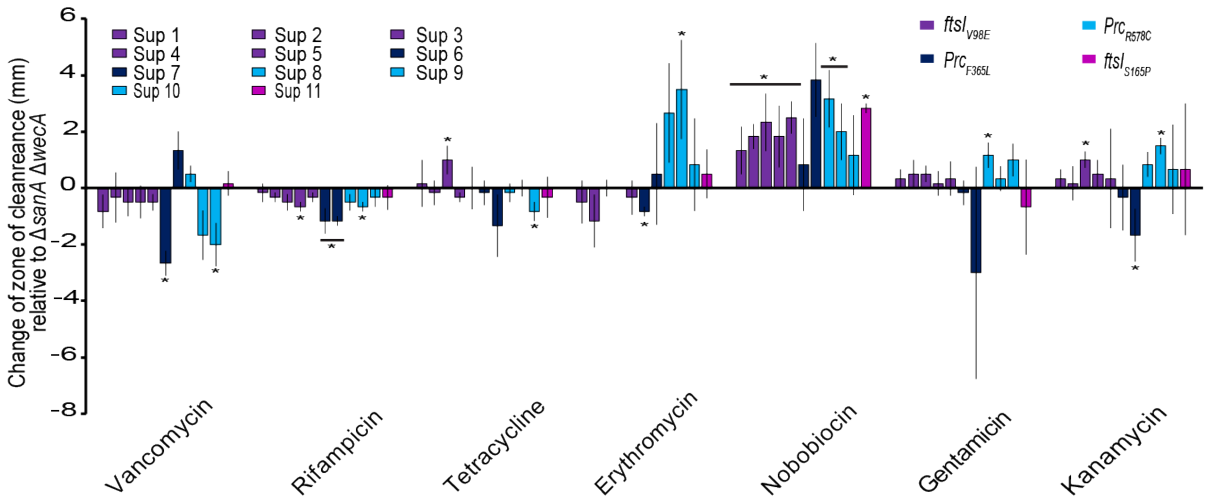

Figure S5: Suppressors have differing antibiotic resistance profiles. The isolated suppressor mutations were assayed by Kirby-Bauer tests for their resistance to a panel of antibiotics. Data are shown as the change in zone of clearance relative to the Δ*sanA* Δ*wecA* strain. For ease of viewing, suppressors have been renumbered and grouped by phenotype. The color of the bars indicates the mutation identified in the indicated strain. Data are the average of three biological replicates ± the SEM.

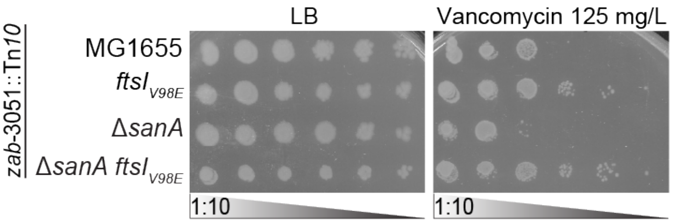

Figure S6: FtsI^{V98E} causes increased vancomycin resistance. EOPs were performed at 43 °C to determine the vancomycin resistance of the indicated strains. The *ftsI*$_{V98E}$ mutation caused increased vancomycin resistance in both the wild-type and Δ*sanA* backgrounds. Images are representative of three independent experiments.

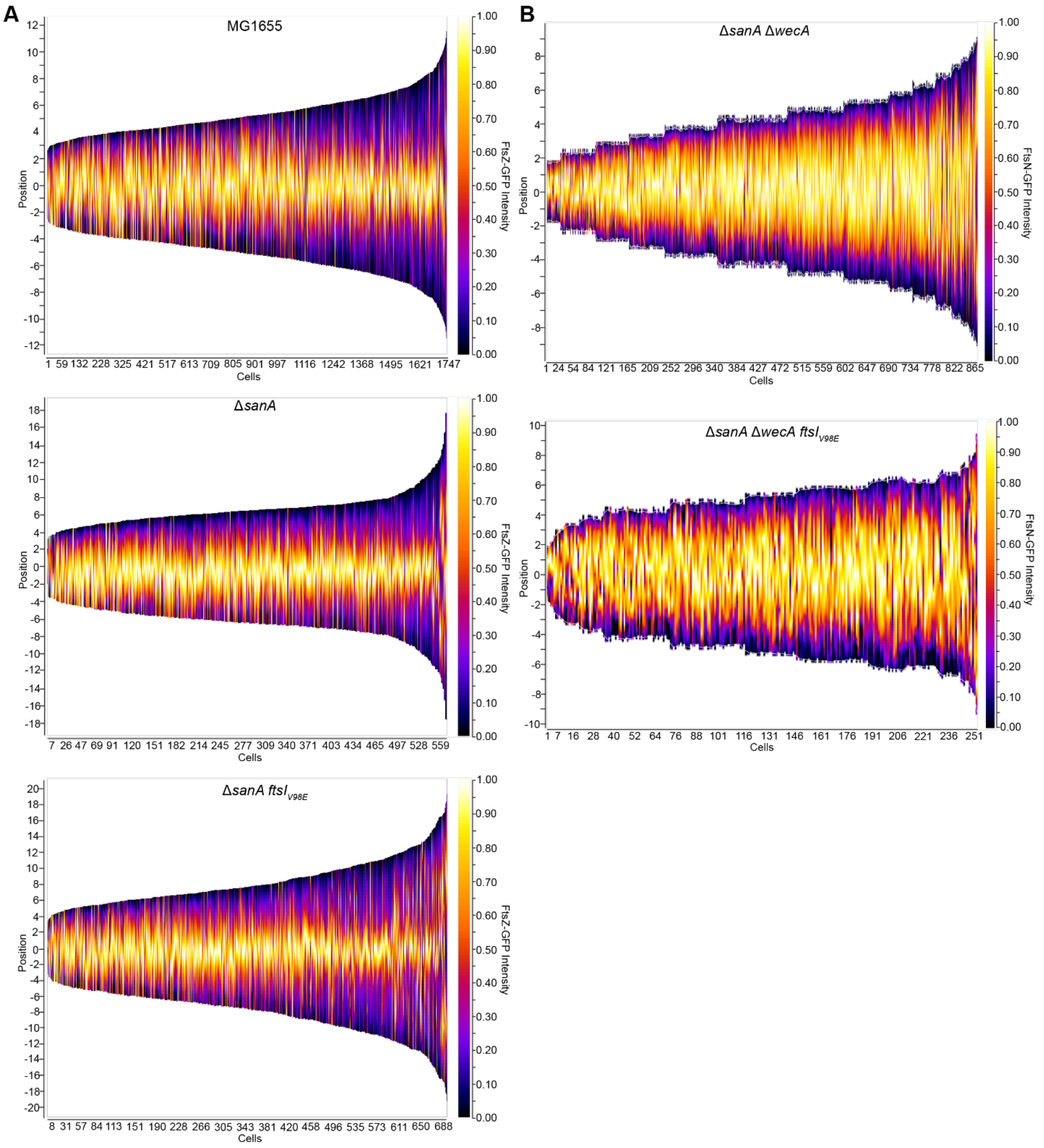

Figure S7: Localization of FtsZ and FtsN do not correlate with phenotypes associated with loss of SanA. (A) The FtsZ-GFP localization was assayed in the wild-type strain, the Δ*sanA* strain, and the Δ*sanA* strain carrying the *ftsI*$_{V98E}$ mutation. Demographs were computed using the locational data with cells arranged from shortest to longest. No significant differences in FtsZ localization were observed. **(B)** FtsN-GFP localization was assessed in the Δ*sanA* Δ*wecA* and Δ*sanA* Δ*wecA ftsI*$_{V98E}$

strains. Demographics were computed using the localization data. No significant differences in FtsN localization were observed.

Table S1: Strains used in this study

Strain	Genotype	Reference	Notes
MG1655	K-12 F⁻ λ⁻ *rph-1*	(1)	CGSC 6300
AM104	MG1655 Δ*rpoS*	(2)	
AM158	MG1655 Δ*sanA*	(2)	
AM202	MG1655 pZS21	This study	Plasmid from (3)
AM203	MG1655 pZS21-*sanA*	This study	
AM213	MG1655 Δ*sanA* pZS21	This study	Plasmid from (3)
AM214	MG1655 Δ*sanA* pZS21-*sanA*	This study	
AM237	MG1655 Δ*yeiS*	This study	
AM334	MG1655 Δ*wecA*	(4)	
AM337	MG1655 Δ*wecE*	(4)	
AM363	MG1655 Δ*mlaC*	This study	
AM380	MG1655 pBR322	This study	Plasmid from (5)
AM381	MG1655 pBR322-*pldA*	This study	Plasmid from (5)
AM382	MG1655 Δ*sanA* pBR322	This study	Plasmid from (5)
AM383	MG1655 Δ*sanA* PBR322-*pldA*	This study	Plasmid from (5)
AM384	MG1655 pBR322-*pldA*	This study	Plasmid from (5)
AM771	MG1655 Δ*sanA* Δ*wecA*	This study	
AM772	MG1655 Δ*sanA* Δ*wecE*	This study	
AM783	MG1655 Δ*wecF*	This study	
AM784	MG1655 Δ*wecG*	This study	
AM785	MG1655 Δ*sanA* Δ*wecF*	This study	
AM786	MG1655 Δ*sanA* Δ*wecG*	This study	
RB006	MG1655 Δ*sanA* Δ*wecA* $ftsI_{V98E}$	This study	Genome nt 91705 T→A
RB007	MG1655 Δ*sanA* Δ*wecA* $ftsI_{S165P}$	This study	Genome nt 91905 T→C
RB010	MG1655 Δ*sanA* Δ*wecA* prc_{F365L}	This study	Genome nt 1913722 G→C
RB011	MG1655 Δ*sanA* Δ*wecA* prc_{R578C}	This study	Genome nt 1913085 G→A
RB036	MG1655 *zab-3051*::Tn*10*	This study	
RB037	MG1655 $ftsI_{V98E}$ *zab-3051*::Tn*10*	This study	
RB038	MG1655 Δ*sanA* $ftsI_{V98E}$ *zab-3051*::Tn*10*	This study	
RB039	MG1655 Δ*wecA* *zab-3051*::Tn*10*	This study	
RB040	MG1655 Δ*wecA* $ftsI_{V98E}$ *zab-3051*::Tn*10*	This study	
RB041	MG1655 Δ*sanA wecA zab-3051*::Tn*10*	This study	
RB044	MG1655 Δ*sanA wecA* $ftsI_{V98E}$ *zab-3051*::Tn*10*	This study	
DW012	MG1655 Δ*sanA wecA* pCH201 (*ftsN*-GFP)	This study	Plasmid from (6)
DW014	MG1655 Δ*sanA wecA ftsI23 leu82*::Tn*10*	This study	*ftsI* allele from (7)
DW017	MG1655 Δ*sanA wecA ftsA* leu82*::Tn*10*	This study	*ftsA* allele from (8)
DW041	MG1655 pXY027	This study	Plasmid from (9)
DW042	MG1655 Δ*sanA* pXY027	This study	Plasmid from (9)
DW043	MG1655 Δ*sanA* Δ*wecA* pXY027	This study	Plasmid from (9)

Strain	Genotype	Reference	Notes
DW068	MG1655 Δ*sanA wecA ftsZ*84 *leu::*Tn*10*	This study	*ftsZ* allele from (10)
DW069	MG1655 Δ*sanA ftsZ*84 *leu::*Tn*10*	This study	*ftsZ* allele from (10)
DW070	MG1655 Δ*sanA ftsA12 leu82::*Tn*10*	This study	*ftsA* allele from (11)
DW071	MG1655 Δ*sanA ftsA27 leu-260::*Tn*10*	This study	*ftsA* allele from (12)
DW072	MG1655 Δ*sanA wecA ftsL* leu82::*Tn*10*	This study	*ftsL* allele from (13)
DW073	MG1655 Δ*sanA wecA ftsQ1 leu82::*Tn*10*	This study	*ftsQ* allele from (14)
DW074	MG1655 Δ*sanA wecA ftsA27 leu-260::*Tn*10*	This study	*ftsA* allele from (12)
DW075	MG1655 Δ*sanA wecA ftsA12 leu82::*Tn*10*	This study	*ftsA* allele from (11)
DW076	MG1655 Δ*sanA ftsL* leu82::*Tn*10*	This study	*ftsL* allele from (13)
DW077	MG1655 Δ*sanA ftsQ1 leu82::*Tn*10*	This study	*ftsQ* allele from (14)
DW081	MG1655 *ftsZ*84 *leu::*Tn*10*	This study	*ftsZ* allele from (10)
DW083	MG1655 *ftsA12 leu82::*Tn*10*	This study	*ftsA* allele from (11)
DW084	MG1655 *ftsA27 leu-260::*Tn*10*	This study	*ftsA* allele from (12)
DW085	MG1655 Δ*ftsW* Δ*araH zab*-3051::Tn*10* pCS37	This study	Plasmid and *ftsW* allele from (15)
DW089	MG1655 Δ*ftsW* Δ*araH zab*-3051::Tn*10* $ftsI_{V98E}$ pCS37	This study	Plasmid and *ftsW* allele from (15)
DW091	MG1655 Δ*sanA* Δ*ftsW* Δ*araH zab*-3051::Tn*10* $ftsI_{V98E}$ pCS37	This study	Plasmid and *ftsW* allele from (15)
DW092	MG1655 Δ*sanA* Δ*wecA* Δ*ftsW* Δ*araH zab*-3051::Tn*10* $ftsI_{V98E}$ pCS37	This study	Plasmid and *ftsW* allele from (15)
DW095	MG1655 Δ*sanA* Δ*wecA* Δ*ftsW* Δ*araH zab*-3051::Tn*10* pCS37	This study	Plasmid and *ftsW* allele from (15)
DW096	MG1655 *ftsL* leu82::*Tn*10*	This study	*ftsL* allele from (13)
DW097	MG1655 *ftsQ1 leu82::*Tn*10*	This study	*ftsQ* allele from (14)
DW099	MG1655 Δ*sanA* Δ*wecA murJ* Ω(−14::*bla araC* P_{BAD}) Δ*araH zab*-3051::Tn*10*	This study	*murJ* allele from (16)
DW0101	MG1655 *murJ* Ω(−14::*bla araC* P_{BAD}) Δ*araH zab*-3051::Tn*10* Δ*sanA*	This study	*murJ* allele from (16)
DW102	MG1655 *murJ* Ω(−14::*bla araC* P_{BAD}) Δ*araH zab*-3051::Tn*10*	This study	*murJ* allele from (16)

Strain	Genotype	Reference	Notes
DW103	MG1655 *murJ* Ω(−14::*bla araC* P_{BAD}) Δ*araH zab*-3051::Tn*10* Δ*sanA* Δ*wecA ftsI*^{V98E}	This study	*murJ* allele from (16)
DW104	MG1655 *murJ* Ω(−14::*bla araC* P_{BAD}) Δ*araH zab*-3051::Tn*10* Δ*sanA ftsI*^{V98E}	This study	*murJ* allele from (16)
DW108	MG1655 Δ*sanA* Δ*wecA zab*-3051::Tn*10 ftsI*^{V98E} pCA24N-*murA*	This study	Plasmid from (17)
DW109	MG1655 Δ*sanA zab*-3051::Tn*10 ftsI*^{V98E} pCA24N-*murA*	This study	Plasmid from (17)
DW110	MG1655 Δ*sanA* Δ*wecA zab*-3051::Tn*10* pCA24N-*murA*	This study	Plasmid from (17)
DW111	MG1655 Δ*sanA zab*-3051::Tn*10* pCA24N-*murA*	This study	Plasmid from (17)
DW112	MG1655 *zab*-3051::Tn*10* pCA24N-*murA*	This study	Plasmid from (17)
DW113	MG1655 *zab*-3051::Tn*10* pCA24N	This study	Plasmid from (17)
DW114	MG1655 Δ*sanA* Δ*wecA zab*-3051::Tn*10* pCA24N	This study	Plasmid from (17)
DW115	MG1655 Δ*sanA zab*-3051::Tn*10* pCA24N	This study	Plasmid from (17)

Table S2: Primers used in this study

Primer	Sequence (5' to 3')
pZS21-RBS-*sanA* F	gtatcgataagcttgatatcaggaggacagctATGTTAAAGCGCGTGTTC
sanA-pZS21 R	gatcccccgggctgcaggaattcCTTTCCTTGTTTCTTTTGTAATTC
pZS21 F	GAATTCCTGCAGCCCGGG
pZS21-RBS R	AGCTGTCCTCCTGATATCAAGCTTATCGATACCGTCGACCTC
sanA-GS-Flag F	tataaagatgatgatgataaatgatgaTCTAGAGGCATCAAATAAAAC
sanA-GS-Flag R	atcgccgctaccccctccagagccaccCTTTCCTTGTTTCTTTTGTAATTC
sanA_V47-G52_R	TGGCGGTAGGGGAGATCC
sanA_V49T_F	GGTCGGTacGGTGCTCGGAACAG
sanA_V50T_F	GGTCGGTGTGacGCTCGGAACAG
sanA_L51N_F	GGTCGGTGTGGTGaaCGGAACAG
sanA_L51A_F	GGTCGGTGTGGTGgcCGGAACAG
sanA_G52S_F	GGTCGGTGTGGTGCTCaGtACAG
sanA_R69_R	TACTGATTAATTACGCCAG
sanA_R69A_F	TTATCGCTACgccATTCAAGGAGC
sanA_R69E_F	TTATCGCTACgaaATTCAAGGAGCG
sanAE98-M102_R	TAACTTTGCAATGCGTTATCGC
sanA_E98A_F	TAATGcGCCGATGACCATGCG
sanA_V111-V117_R	CCAGCAGCGATTAAATCTTTG
sanA_I116S_F	TGTCGACCCATCAGATtcTGTTC
sanA_L118-L126_R	CAATATCTGATGGGTCGACACC
sanA_L118N_F	TTaatGATTACGCAGGCTTTCGTACGCTGGA
sanA_V130-V135_R	AGCGTACGAAAGCCTGCG
sanA_V130T_F	GGATTCCATCacgCGTACACGCAAAGTTTTC
sanA_V135T_F	GGATTCCATCGTGCGTACACGCAAAacTTTC
sanA_R131-F136_R	ACGATGGAATCCAGCGTAC
sanA_F136Q_F	GCGTACACGCAAAGTTcaaGATA
sanA_T145_R	AAATCATTAGTATCGAAAACTTTG
sanA_T145A_F	CATTATTATCgctCAACGTTTCC
sanA_T145N_F	CATTATTATCaatCAACGTTTCCACTGTG
sanA_H149-R152_R	CGTTGGGTGATAATAATGAAATCATTAG
sanA_H149A_F	TTTCgccTGTGAGCGAGCATT
sanA_R152A_F	TTTCCACTGTGAGgcaGCATT
sanA_R152E_F	TTTCCACTGTGAGgaaGCATT
sanA_L176_R	GGTGACGGTACGGCATAA
sanA_L176N_F	GAAAGATATGaatTCAGTACGTATTCGTGAATTTGCC
sanA_I180-F183_R	ACTGACAGCATATCTTTC
sanA_E182R_F	ACGTATTCGTaggTTTGCCGCCC
sanA_F183Q_F	ACGTATTCGTGAAcaaGCCGCCC
sanA_D192_R	CCGAAACGGGCGGCAAAT
sanA_D192R_F	TGCGCTGGCTcgcCTTTATATTTTTAAACGTG
sanA_R198_R	TAAAGGTCAGCCAGCGCA
sanA_R198D_F	TATTTTTAAAgacGAACCGCGTTTTTTAGGGCCGC
sanA_D192R_R	TAAAGgcgAGCCAGCGCA

Supplemental References

1. Guyer MS, Reed RR, Steitz JA, Low KB. 1981. Identification of a sex-factor-affinity site in E. coli as gamma delta. Cold Spring Harb Symp Quant Biol 45 Pt 1:135-40.
2. Mitchell AM, Wang W, Silhavy TJ. 2017. Novel Rpos-dependent mechanisms strengthen the envelope permeability barrier during stationary phase. J Bacteriol 199:e00708-16.
3. Lutz R, Bujard H. 1997. Independent and tight regulation of transcriptional units in *Escherichia coli* via the LacR/O, the TetR/O and AraC/I1-I2 regulatory elements. Nucleic Acids Res 25:1203-10.
4. Mitchell AM, Srikumar T, Silhavy TJ. 2018. Cyclic enterobacterial common antigen maintains the outer membrane permeability barrier of *Escherichia coli* in a manner controlled by Yhdp. mBio 9:e01321-18.
5. Malinverni JC, Silhavy TJ. 2009. An ABC transport system that maintains lipid asymmetry in the Gram-negative outer membrane. Proceedings of the National Academy of Sciences 106:8009-8014.
6. Hale CA, de Boer PA. 2002. ZipA is required for recruitment of FtsK, FtsQ, FtsL, and FtsN to the septal ring in *Escherichia coli*. J Bacteriol 184:2552-6.
7. Begg KJ, Donachie WD. 1985. Cell shape and division in *Escherichia coli:* experiments with shape and division mutants. J Bacteriol 163:615-22.
8. Geissler B, Elraheb D, Margolin W. 2003. A gain-of-function mutation in *ftsA* bypasses the requirement for the essential cell division gene *zipA* in *Escherichia coli*. Proceedings of the National Academy of Sciences 100:4197-4202.
9. Buss J, Coltharp C, Shtengel G, Yang X, Hess H, Xiao J. 2015. A multi-layered protein network stabilizes the *Escherichia coli* FtsZ-ring and modulates constriction dynamics. PLoS Genet 11:e1005128.
10. Hirota Y, Ryter A, Jacob F. 1968. Thermosensitive mutants of E. coli affected in the processes of DNA synthesis and cellular division. Cold Spring Harb Symp Quant Biol 33:677-93.
11. Wijsman HJ, Koopman CR. 1976. The relation of the genes envA and ftsA in Escherichia coli. Mol Gen Genet 147:99-102.
12. Dai K, Xu Y, Lutkenhaus J. 1993. Cloning and characterization of *ftsN*, an essential cell division gene in *Escherichia coli* isolated as a multicopy suppressor of *ftsA12*(Ts). J Bacteriol 175:3790-7.
13. Ishino F, Jung HK, Ikeda M, Doi M, Wachi M, Matsuhashi M. 1989. New mutations fts-36, lts-33, and ftsW clustered in the mra region of the Escherichia coli chromosome induce thermosensitive cell growth and division. J Bacteriol 171:5523-30.
14. Begg KJ, Hatfull GF, Donachie WD. 1980. Identification of new genes in a cell envelope-cell division gene cluster of Escherichia coli: cell division gene ftsQ. J Bacteriol 144:435-7.
15. Sham L-T, Butler EK, Lebar MD, Kahne D, Bernhardt TG, Ruiz N. 2014. MurJ is the flippase of lipid-linked precursors for peptidoglycan biogenesis. Science 345:220-222.

16. Ruiz N. 2008. Bioinformatics identification of MurJ (MviN) as the peptidoglycan lipid II flippase in *Escherichia coli*. Proceedings of the National Academy of Sciences 105:15553-15557.
17. Kitagawa M, Ara T, Arifuzzaman M, Ioka-Nakamichi T, Inamoto E, Toyonaga H, Mori H. 2005. Complete set of ORF clones of *Escherichia coli* ASKA library (a complete set of *E. coli* K-12 ORF archive): unique resources for biological research. DNA Res 12:291-9.
